# NPR-1 Modulates Plasticity in *C*. *elegans* Stress-Induced Sleep

**DOI:** 10.1016/j.isci.2019.08.050

**Published:** 2019-08-30

**Authors:** Rony Soto, Desiree L. Goetting, Cheryl Van Buskirk

**Affiliations:** 1 Department of Biology, California State University Northridge, Northridge, CA 91330, USA

**Keywords:** Physiology, Genetics, Behavioral Neuroscience

## Abstract

Sleep is beneficial yet antagonistic to critical functions such as foraging and escape, and we aim to understand how these competing drives are functionally integrated. *C*. *elegans*, which lives in reduced oxygen environments, engages in developmentally timed sleep (DTS) during larval stage transitions and engages in stress-induced sleep (SIS) during recovery from damaging conditions. Although DTS and SIS use distinct mechanisms to coordinate multiple sleep-associated behaviors, we show that movement quiescence in these sleep states is similarly integrated with the competing drive to avoid oxygen. Furthermore, by manipulating oxygen to deprive animals of SIS, we observe sleep rebound in a wild *C*. *elegans* isolate, indicating that sleep debt accrues during oxygen-induced SIS deprivation. Our work suggests that multiple sleep states adopt a common, highly plastic effector of movement quiescence that is suppressed by aversive stimuli and responsive to homeostatic sleep pressure, providing a limited window of opportunity for escape.

## Highlights

• NPR-1 promotes locomotor quiescence during stress-induced sleep (SIS)
• Arousal from SIS in *npr-1(lf)* is partly dependent on PDF secretion
• Oxygen levels dynamically influence SIS
• Sleep rebound is observed after SIS deprivation

## Graphical Abstract

**Figure:**
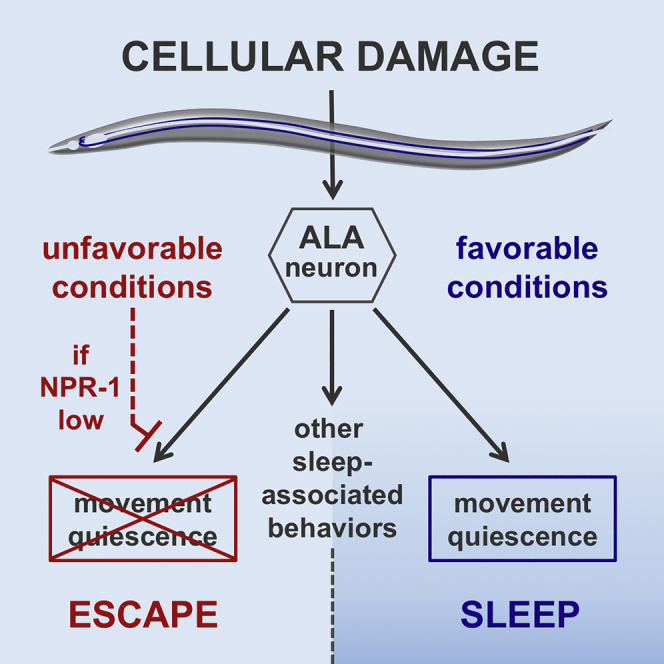

## Introduction

Animals from cnidarians to vertebrates engage in sleep, quickly reversible periods of behavioral quiescence that are associated with reduced sensory responsiveness and homeostatic regulation (reviewed in Keene and Duboue, 2018, Anafi et al., 2019). Although the cellular function of sleep is of debate, its benefit is inarguable, and sleep loss is associated with a range of deleterious consequences (reviewed in Cirelli and Tononi, 2008). Sleep precludes the execution of critical functions such as foraging or escape, and there are likely mechanisms that coordinate these competing drives at multiple levels. The circadian regulation of sleep may represent one such level, restricting sleep to times when the effectiveness of foraging and the danger of predation are reduced. Mechanisms that provide behavioral plasticity in sleep regulation in response to more unpredictable conditions are beginning to be elucidated. For example, a peptide released by *Drosophila* males during copulation suppresses daytime sleep in females, allowing increased foraging and egg-laying (Isaac et al. 2010). Starvation has long been known to suppress sleep across species, including mammals (Jacobs and McGinty, 1971, MacFayden et al., 1973), and recent work in *Drosophila* and *C*. *elegans* has shed light on the molecular basis of this effect (Keene et al., 2010, Goetting et al., 2018, Yurgel et al., 2019).

Two sleep states have been described in *C*. *elegans*, neither of which falls under circadian regulation. This nematode sleeps at the end of each larval molt during a period known as developmentally timed sleep (DTS) or lethargus (Raizen et al., 2008) and also during recovery from exposure to damaging conditions, a phenomenon referred to as stress-induced sleep (SIS) (Hill et al., 2014, Nelson et al., 2014). SIS is triggered by exposure to damaging agents such as UV light and heat and is dependent on Epidermal Growth Factor (EGF) signaling and ALA interneuron function. ALA promotes a coordinated quiescent state through the collective action of neuropeptides that impact an array of sleep sub-behaviors (Hill et al., 2014, Nelson et al., 2014, Iannacone et al., 2017, Nath et al., 2016). Engagement in SIS appears to be beneficial, as sleep-defective mutants are impaired for recovery following exposure to damaging conditions (Hill et al., 2014, Fry et al., 2016). Interestingly, a robust SIS-like state can be induced in the absence of damaging conditions through transient overexpression of LIN-3/EGF, an effect that requires EGF receptor function in the ALA neuron (Van Buskirk and Sternberg, 2007). DTS, while phenotypically similar to SIS, is genetically and neurochemically distinguishable (Trojanowski et al., 2015). DTS is linked to the molting cycle by the activity of the PERIOD homolog LIN-42 (Monsalve et al., 2011) and is largely intact in ALA-ablated animals (Van Buskirk and Sternberg, 2007). Head movement quiescence in both SIS and DTS depends on the RIS interneuron (Turek et al., 2013, Grubbs et al., 2019, Konietzka et al., 2019, Robinson et al., 2019), indicating that the two sleep programs utilize a common effector of quiescence for at least one active behavior.

The neurogenetic tractability of *C*. *elegans* DTS and SIS provides attractive models for the examination of mechanisms that confer plasticity in sleep regulation. The *C*. *elegans* neuropeptide Y receptor homolog NPR-1 influences responsiveness to a variety of external cues, including oxygen (Gray et al., 2004), pheromones (Macosko et al., 2009), and mechanical stimuli (Choi et al., 2013). The laboratory strain N2 harbors a high-activity NPR-1 variant and shows robust DTS under a wide range of conditions. By contrast, N2-derived *npr-1* loss-of-function (lf) mutants as well as wild isolates with low NPR-1 activity engage in DTS only under conditions that minimize arousing cues (Choi et al., 2013, Nagy et al., 2014, Nichols et al., 2017). DTS is associated with global changes in neural activity (Nichols et al., 2017), and locomotor arousal from DTS in *npr-1* mutants is partly dependent on heightened activity of the RMG circuit (Choi et al., 2013, Nichols et al., 2017) and, in some circumstances, secretion of the wake-promoting peptide PDF-1 acting on receptors within touch neurons (Choi et al., 2013). These studies indicate that movement quiescence is a highly plastic component of DTS that is sensitive to NPR-1 function. The role of NPR-1 in regulating DTS has been well characterized, whereas its role in SIS, if any, has not been determined.

In a genetic screen for SIS-defective mutants, we have uncovered *csn7*, an allele of *npr-1*. We show that *npr-1*(*csn7*) and other *npr-1*(*lf*) mutants engage in SIS when aggregating but not when solitary. The quiescence defect is specific to locomotion, with feeding quiescence intact, and is observed during both SIS and EGF-induced sleep. We find the locomotor arousal of *npr-1* mutants during SIS to depend partly on the wake-promoting Pigment-Dispersing Factor PDF-1. We show that SIS in *npr-1(lf)* animals is rapidly responsive to changes in ambient oxygen and that the SIS defect observed under normoxic conditions is suppressed by mutations that interfere with oxygen sensation. Last, we use the wake-promoting effect of oxygen to deprive animals of SIS and observe sleep rebound in a wild (low NPR-1 activity) *C*. *elegans* isolate. Our results reveal that oxygen avoidance can dynamically override SIS and that sleep debt accrues during oxygen-induced SIS deprivation.

## Results

**Figure 1:**
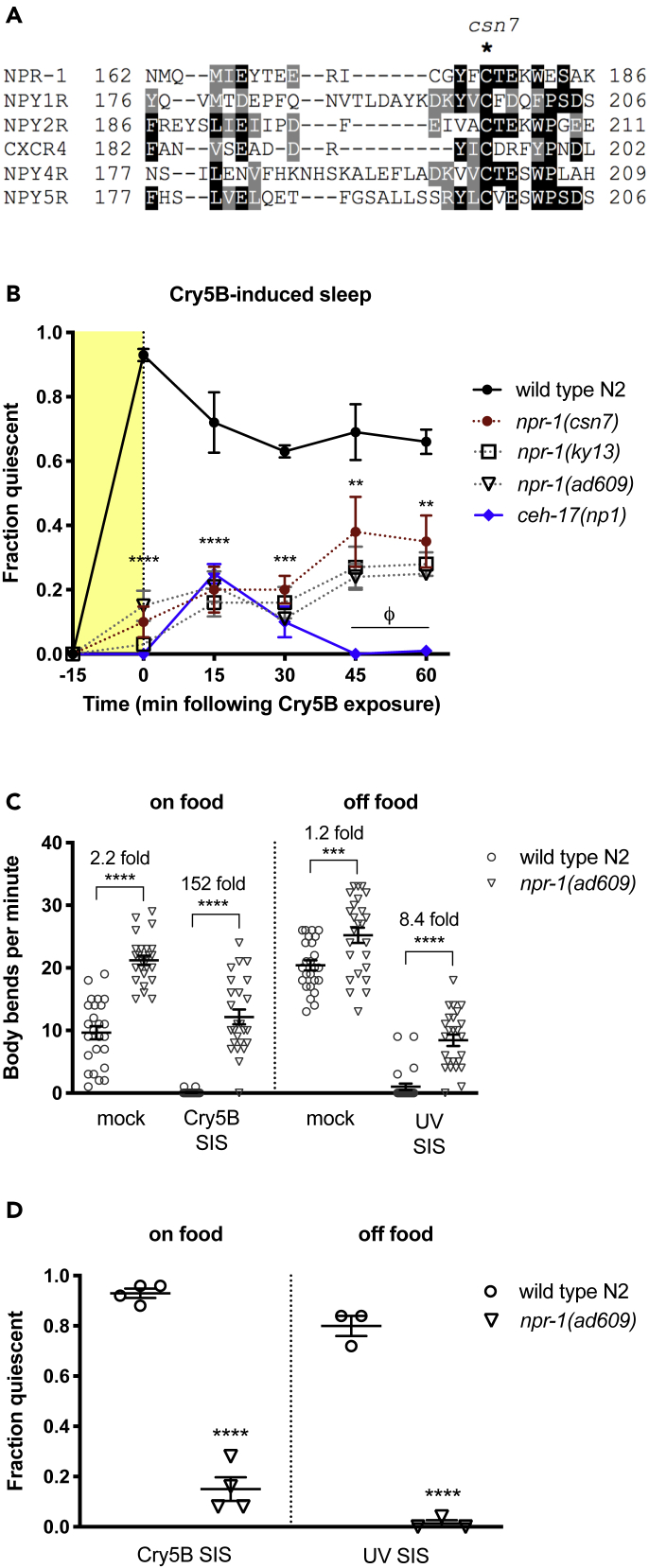
*csn7* Is an Allele of *npr-1* (A) The protein sequence of the second predicted extracellular loop of NPR-1 (de Bono and Bargmann, 1998) is shown, aligned with human NPY receptors. *csn7* harbors a Tyrosine substitution for the conserved Cys178. T-Coffee Multiple Sequence Alignment and BoxShade visualization tool. (B) *npr-1* mutants are defective in stress-induced sleep following exposure to Cry5B pore-forming toxin. Animals were exposed to Cry5B toxin for 15 min (shading) and examined for behavioral quiescence, defined as cessation of all movement and feeding. The majority of quiescence following Cry5B exposure is ALA dependent, as is evidenced by ALA-impaired *ceh-17* mutants. *npr-1*(*csn7*) and the *npr-1*(*lf*) alleles *ad609* and *ky13* initially show SIS defects similar to *ceh-17*(*lf*) but over time engage in partially penetrant SIS. At least three trials of 25 young adult hermaphrodites were performed for each genotype. Mean and SEM are shown. **p < 0.01, ***p < 0.001, ****p < 0.0001 *csn-7* versus wild-type; φ p < 0.0001 *npr-1(ad609)* versus *ceh-17(np1)*, two-way repeated measures (RM) ANOVA with Sidak's multiple comparisons test. (C and D) *npr-1(lf)* arousal during SIS is robust to variations in baseline locomotor activity. (C) Comparison of wild-type and *npr-1(lf)* locomotion during and outside of SIS. For Cry5B-SIS, animals were exposed to either *E*. *coli* OP50 (mock controls) or Cry5B-expressing *E*. *coli* for 15 min before examination. For UV-SIS in the absence of food, animals were rinsed and transferred to NGM plates lacking peptone and bacteria, exposed to UV for 1 min or mock treated, and examined 60 min later. Data points indicate individual animals. Mean and SEM are shown. ***p < 0.001, ****p < 0.0001, one-way ANOVA with Sidak's multiple comparisons test. (D) Striking differences in SIS are seen between wild-type and *npr-1(lf)* animals under conditions with large (on food) or small (off food) differences in baseline locomotion. Each data point represents the fraction of quiescent animals (no feeding, no locomotion) in one trial of 25 young adult hermaphrodites. Mean and SEM of multiple independent trials are indicated. ****p < 0.0001 versus wild-type, Student's t test.

To identify genes regulating SIS in *C*. *elegans*, we performed a non-clonal EMS screen for mutants that fail to engage in SIS following ingestion of the pore-forming toxin Cry5B, which triggers a robust bout of sleep that is dependent on the ALA neuron (Hill et al., 2014). This primary screen was followed by secondary screening for resistance to other conditions known to trigger ALA-dependent sleep, including noxious heat and UV light. In this screen we recovered *csn7*. In addition to its SIS defect, we noted that *csn7* animals aggregated in a manner reminiscent of animals with reduced function of NPR-1, a G-protein-coupled receptor related to mammalian neuropeptide Y (NPY) receptors (de Bono and Bargmann, 1998). Complementation analysis and sequencing of the *npr-1* gene in the *csn7* mutant revealed *csn7* to be an allele of *npr-1*. The *npr-1(csn7)* mutant harbors a G-to-A transition at nucleotide 533 of the NPR-1 coding sequence, resulting in a Cys178Tyr substitution. Cys178 lies in the extracellular region between predicted transmembrane domains 4 and 5 (de Bono and Bargmann, 1998) and is conserved in human NPY receptors (Figure 1A). We examined the previously characterized null *npr-1* alleles *ad609* and *ky13* (de Bono and Bargmann, 1998) for defects in SIS. Animals were exposed to Cry5B toxin and examined for behavioral quiescence, defined by cessation of both movement and feeding. We and others have shown that behavioral quiescence during SIS is associated with hallmarks of sleep including reduced sensory responsiveness and quick reversibility (Hill et al., 2014, DeBardeleben et al., 2017). We observed a similar SIS defect in all *npr-1* alleles examined (Figure 1B). The phenotypic similarity between *csn7* and presumptive null alleles, along with the substitution of a highly conserved amino acid, suggests that *csn7* is a loss-of-function allele. In contrast to *ceh-17* mutants that lack the function of the sleep-inducing ALA neuron, *npr-1* mutants eventually engage in partially penetrant SIS, suggesting that the sleep pathway is intact but that the ability to engage in quiescent behavior is transiently suppressed.

Loss of *npr-1* is known to be associated with accelerated locomotion in the presence of food (de Bono and Bargmann, 1998), raising the possibility that the sleep defect in these animals is a secondary consequence of increased baseline locomotor activity. We investigated this possibility in two ways. First, we examined the impact of NPR-1 on locomotor activity during and outside of SIS and found that loss of NPR-1 had a considerably larger effect on locomotion during SIS (152-fold increase in body bend rate compared with wild-type N2) than outside of SIS (2-fold increase in body bend rate) (Figure 1C). Second, we examined SIS in the absence of food, a condition that nearly eliminates the difference in baseline locomotion conferred by NPR-1 activity (de Bono and Bargmann, 1998; Figure 1C). In this assay, UV light exposure (DeBardeleben et al., 2017) was used rather than Cry5B toxin to trigger SIS, as this assay can be performed in the absence of bacteria. Under these conditions, SIS is still highly dependent on NPR-1 (Figure 1D). Thus, increased baseline locomotor activity cannot account for the failure of *npr-1* mutants to engage in movement quiescence during SIS. These results reveal a previously unknown role for NPR-1 in SIS.

### NPR-1 Promotes Locomotor Quiescence during SIS

**Figure 2:**
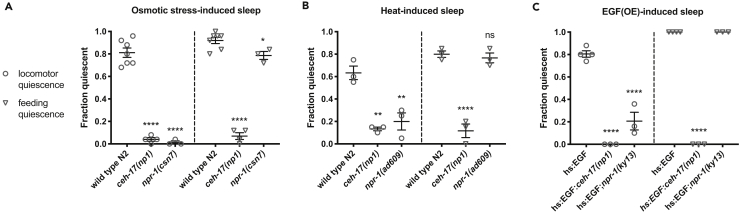
NPR-1 Modulates Locomotor Arousal during SIS and EGF-Induced Sleep (A and B) NPR-1 is required for locomotor quiescence during SIS induced by osmotic stress (A) and noxious heat (B). Young adult animals were exposed to stressors as described in methods and examined for locomotion and feeding during ALA-dependent sleep, as indicated by comparison of wild-type N2 and *ceh-17(np1)* ALA-impaired animals. (C) NPR-1 is required for locomotor quiescence during EGF-induced sleep. Overexpression of LIN-3/EGF in the wild-type background results in cessation of locomotion and feeding, effects that are CEH-17 dependent. Animals were examined 2 h following transgene induction. Each data point represents the fraction of quiescent animals in one trial of 25 young adult hermaphrodites. Mean and SEM of multiple independent trials are indicated. ns, not significant, *p < 0.05, **p < 0.01, ****p < 0.0001 versus control, one-way ANOVA with Dunnett's multiple comparisons test.

SIS depends on the coordinated activity of several ALA-derived neuropeptides with distinct but overlapping functions in the inhibition of locomotion, feeding, and other behaviors (Nath et al., 2016). As such, mutations such as *ceh-17* that impair ALA function produce coordinated sleep defects (Hill et al., 2014), whereas mutations affecting tissue-specific responses to ALA neuropeptides are expected to impact specific sub-behaviors of sleep. To characterize the relative contribution of NPR-1 to locomotor and feeding quiescence during adult SIS, we quantified each of these behaviors in *npr-1* mutants. As ingestion of Cry5B and exposure to UV light have ALA-independent effects on pharyngeal pumping (Hill et al., 2014, Goetting et al., 2018), we assayed heat-induced and salt-induced sleep, for which ALA-independent effects are transient (Hill et al., 2014). The *ceh-17(lf)* mutants are severely impaired for both locomotor and feeding quiescence, whereas *npr-1(lf)* mutants are specifically defective in locomotor quiescence during SIS (Figures 2A and 2B). This differential impact on the sub-behaviors of SIS suggests that NPR-1 impacts SIS downstream of ALA function, ruling out a major role in modulating sensitivity to cellular damage. To confirm this, we activated the ALA neuron in the absence of damaging conditions, by overexpressing (OE) LIN-3/EGF (Van Buskirk and Sternberg, 2007). We found that locomotor quiescence during EGF(OE)-induced sleep is dependent on NPR-1 (Figure 2C), suggesting that NPR-1 functions downstream of, or in parallel to, ALA activation to modulate locomotor arousal during SIS.

### PDF-1 Contributes to SIS Arousal in *npr-1* Mutants

*C*. *elegans* DTS (also known as lethargus) occurs during the period of cuticle restructuring that precedes each larval molt (Raizen et al., 2008). Interestingly, NPR-1 has been shown to be required for locomotor quiescence during DTS (Choi et al., 2013, Nichols et al., 2017). Locomotor arousal in *npr-1(lf)* during DTS has been associated with heightened sensitivity to touch (Choi et al., 2013, Nagy et al., 2014) and to oxygen (Nichols et al., 2017). We wished to examine the contribution of these cues to the arousal of *npr-1(lf)* during SIS. We first examined the contribution of touch sensitivity.

**Figure 3:**
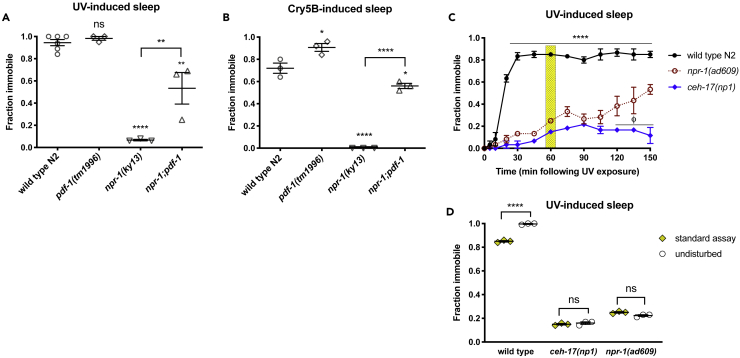
The SIS Defect in *npr-1(lf)* Is Influenced by PDF-1 Secretion but Is Not Attributable to Heightened Mechanosensation (A and B) Animals were assayed for SIS in response to UV light (A) or Cry5B toxin (B) as described in methods. Each data point represents the fraction of immobile animals in one trial of 25 young adult hermaphrodites. Mean and SEM of multiple independent trials are indicated. *p < 0.05, **p < 0.01, ****p < 0.0001, ns, not significant versus Wild-type (or versus genotype indicated by connecting bars), one-way ANOVA with Sidak's multiple comparisons test. (C) Time course of locomotor quiescence following UV light exposure. In this standard assay, plates are gently moved into the stereomicroscope field of view 45 s before each time point. *npr-1(lf)* animals are defective in locomotor quiescence following UV exposure, similar to ALA-defective *ceh-17(lf)* but ultimately engage in partially penetrant SIS. Mean and SEM of three trials of at least 20 animals per trial are shown. ****p < 0.0001 wild-type versus *npr-1(ad609)*, φ p < 0.01 *npr-1(ad609)* versus *ceh-17(np1)*, two-way RM ANOVA with Sidak's multiple comparisons test. (D) The SIS defect of *npr-1(lf)* does not appear to be attributable to heightened touch sensitivity. The 60 min time point from the time course in (C) (yellow shading) is compared with that of single-time point assay in which plates are left completely undisturbed 60 min before examination. Wild-type, but not *npr-1(lf)*, animals show increased locomotor quiescence in response to reduced mechanosensory input. Each data point represents the fraction of immobile animals in one trial of at least 20 young adult hermaphrodites. Mean and SEM of multiple independent trials are indicated. ****p < 0.0001, ns, not significant, multiple Student's t tests with Holm-Sidak correction.

Arousal of *npr-1(lf)* animals from DTS has been found to be dependent on secretion of PDF-1, an arousal peptide acting via receptors in peripheral mechanosensory neurons and body muscles, possibly increasing the sensitivity of these tissues to stimulation (Choi et al., 2013). To determine whether PDF-1 is required for locomotor arousal of *npr-1(lf)* during SIS, we examined *npr-1*;*pdf-1* double mutant animals. We found that *pdf-1(lf)* reduces but does not eliminate arousal from SIS in *npr-1(lf)* (Figures 3A and 3B), suggesting that the wake-promoting activity of PDF-1 contributes to the SIS defect associated with loss of NPR-1 function. We also examined the contribution of touch sensitivity to locomotor arousal by reducing the level of mechanosensory input from plate movement and from animal interactions. In this assay, we examined UV-SIS, as the SIS response to UV exposure is long-lasting (DeBardeleben et al., 2017) and animals can be left completely unperturbed for a long period before examination of behavior. We compared the SIS responses of animals in our standard assay (25 animals per plate, gently moved on the microscope stage every 10 min; Figure 3C) and the “undisturbed” assay (five animals per plate, undisturbed for 60 min before examination). Wild-type animals responded to the undisturbed assay with increased quiescence (Figure 3D), indicating that this difference in mechanosensory input is capable of impacting SIS. By contrast, the quiescence of *npr-1(lf)* animals did not increase under the same conditions (Figure 3D). We interpret these results to indicate that the SIS defect of *npr-1* mutants is not attributable to heightened touch sensitivity.

### Hyperoxia Avoidance Dynamically Overrides Stress-Induced Sleep

**Figure 4:**
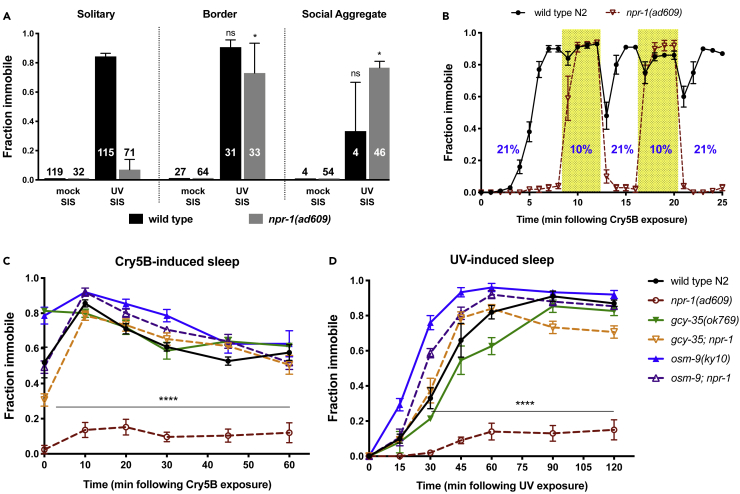
Hyperoxia Avoidance Suppresses SIS in *npr-1* Mutants (A) *npr-1(lf)* animals are more likely to engage in SIS when bordering or aggregating than when solitary. Animals were exposed to UV light or mock treated and 60 min later categorized as solitary, bordering, or aggregating as described in methods and examined for locomotor quiescence. Wild-type animals rarely aggregate, hence the low *n* for this category. Mean and SEM of three trials of 50 animals per trial are shown, and the total number of animals in each category is indicated. *p < 0.05, ns, not significant versus solitary animals of same genotype, one-way ANOVA with Dunnett's multiple comparisons test. For a time course of bordering and aggregation behavior during SIS, see Figure S1. (B) Wild-type N2 and *npr-1(lf)* animals were exposed to Cry5B for 10 min, transferred to plates containing *E*. *coli* OP50, placed into a Hammond chamber, and exposed to controlled oxygen conditions as described in methods. *npr-1(lf)* are sustainably aroused by ambient oxygen (21%) but quickly become quiescent in response to low oxygen (10%). (C and D) Genetic components of oxygen sensation are required for arousal of *npr-1(lf)* animals from SIS. Animals were exposed to either Cry5B (C) or UV light (D) as SIS triggers and examined for locomotor quiescence. Impairment of the hyperoxia avoidance response, using *gcy-35(ok769) or osm-9(ky10)*, abrogates the SIS defect of *npr-1(ad609)* animals. Mean and SEM of three trials of at least 25 young adult animals per trial are shown. ****p < 0.0001 double mutants versus *npr-1(ad609)*, two-way RM ANOVA with Sidak's multiple comparisons test.

We next examined the contribution of hyperoxia avoidance to the arousal of *npr-1(lf)* from SIS. Animals with reduced NPR-1 function are hypersensitive to oxygen (Gray et al., 2004) and seek lower-oxygen environments such as the thickened border of bacterial lawns and “social aggregates” of worms (de Bono and Bargmann, 1998). Under these and other low-oxygen conditions, *npr-1(lf)* animals are competent to engage in DTS (Nichols et al., 2017). To investigate whether the *npr-1* SIS defect may be attributable to oxygen avoidance, we first examined whether *npr-1* mutants are more likely to engage in SIS when bordering and/or aggregating than when they are solitary on the open bacterial lawn. We found the fraction of animals engaging in SIS to be increased among animals in the lawn border or in social aggregates than when solitary on the open lawn (Figure 4A). This effect is not attributable to baseline differences in mobility under these conditions, as evidenced by mock SIS-treated controls (Figure 4A).

Second, we examined SIS behavior of *npr-1(lf)* animals under controlled reduced-oxygen conditions (10% oxygen) and found that this potently suppressed locomotor arousal (Figure 4B). This effect is strikingly rapid, penetrant, sustainable, and quickly reversible: a large fraction of sleepless *npr-1(lf)* animals become completely immobile within 15 s of oxygen reduction, an effect that is reversed equally rapidly upon exposure to normoxic conditions. A fraction of wild-type animals are aroused by the transition between oxygen concentrations, but this effect is very transient.

Last, we examined whether disruption of the oxygen avoidance circuit could restore wild-type SIS behavior to *npr-1(lf)* mutants. Oxygen avoidance contributes to aggregation behavior in *npr-1(lf)*, which is dependent on heightened activity of the RMG interneuron (Macosko et al., 2009), the hub of an electrically coupled circuit including oxygen-sensing and other sensory neurons. Mutations in the O_2_-sensing soluble guanylate cyclase encoded by *gcy-35* abolish oxygen avoidance behaviors of *npr-1(lf)* (Gray et al., 2004, Cheung et al., 2005, Chang et al., 2006), as do mutations in *osm-9*, encoding a subunit of a transient receptor potential vanilloid (TRPV) channel subunit required for sensory transduction in the RMG circuit (de Bono et al., 2002, Chang et al., 2006, Choi et al., 2013, Laurent et al., 2015). We found that mutations in each of these genes restored SIS behavior to *npr-1(ad609)* mutants (Figures 4C and 4D). Together, these data indicate that an oxygen avoidance circuit, including primary oxygen sensors and most likely the RMG interneurons, overrides movement quiescence during SIS in a rapid and reversible manner.

### NPR-1 Variation Contributes to SIS Variation between Polymorphic Strains

**Figure 5:**
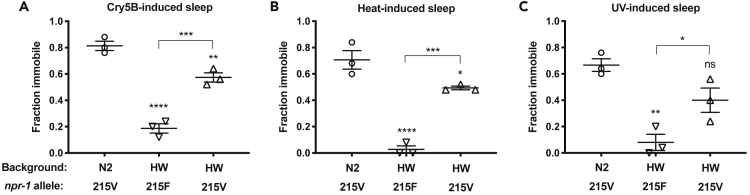
A High-Activity NPR-1 Variant Partially Restores Stress-Induced Sleep to the Polymorphic Hawaii Strain (A–C) Animals were exposed to SIS triggers as indicated and examined for locomotor quiescence as described in methods. Compared with the laboratory N2 strain, the Hawaii (HW) wild isolate fails to engage in locomotor quiescence during SIS. Locomotor quiescence is restored partially by introgression of the N2 NPR-1 genomic region into the Hawaii background (strain CX11400). Each data point represents the fraction of immobile animals in one trial of 25 young adult animals. Mean and SEM of three independent trials are shown. *p < 0.05, **p < 0.01, ***p < 0.001, ****p < 0.0001, ns, not significant versus N2 (unless otherwise indicated by connecting bars on graphs), one-way ANOVA with Tukey's multiple comparisons test.

Naturally occurring polymorphic isolates of *C*. *elegans*, including the Hawaiian strain CB4856, carry a lower-activity variant of NPR-1 (215F) than the laboratory strain N2 (NPR-1 215V) (de Bono and Bargmann, 1998). We wished to examine whether these strains differ in their SIS responses. We examined the Hawaiian isolate under standard laboratory conditions and found that this strain had a reduced SIS response compared with N2 (Figures 5A–5C). To determine whether variation in the *npr-1* gene is a major component of the difference in SIS behavior between the N2 and Hawaii strains, we examined a strain containing a genomic segment (<200 kb) containing the NPR-1(215V) variant from N2 introgressed into the Hawaii genetic background (Bendesky, 2012). We found that the replacement of the low-activity NPR-1(215F) with the 215V variant partially restored SIS locomotor quiescence to the wild strain. Thus the N2 and Hawaii polymorphic strains differ in their SIS responses, and some but not all of this difference is attributable to variation in NPR-1.

### SlS May Be Homeostatically Regulated

**Figure 6:**
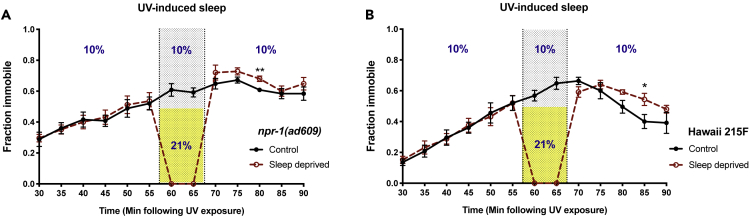
Increased Quiescence Is Observed Following Oxygen-Induced SIS Deprivation (A and B) Animals were exposed to UV light, placed into a Hammond chamber, and exposed to low oxygen (10%) as described in methods to allow animals to engage in SIS. One of the two plates was removed from the chamber and exposed to 21% oxygen (yellow shading) for sleep deprivation, followed by return to low oxygen (sleep-permissive) conditions. In *npr-1(lf)* mutant animals (A) and the Hawaiian wild isolate (B), sleep-deprived animals show increased locomotor quiescence relative to control animals. Mean and SEM of five independent trials of 25 animals each are shown. *p < 0.05, **p < 0.01, sleep-deprived versus control, Student's t test.

The homeostatic regulation of sleep is apparent when periods of sleep deprivation are followed by increased sleep relative to non-deprived subjects. Homeostatic regulation has been observed in *C*. *elegans* DTS (Raizen et al., 2008, Nagy et al., 2014, Nichols et al., 2017), whereas it has not yet been observed in the context of SIS. We used oxygen arousal to control the duration of SIS deprivation in both *npr-1(lf)* and in the Hawaiian isolate CB4856 harboring a low-activity NPR-1 variant (215F). Following exposure to UV light, the exposed population was separated to two plates and placed in a low-oxygen chamber to allow animals to engage in SIS. After 55 min, one plate was removed to 21% oxygen for sleep deprivation and the control plate was returned to low-oxygen conditions. After 10 min the sleep-deprived animals were returned to the chamber and low-oxygen conditions were re-established. During each transition the control group experienced a maximum of 2 min of sleep deprivation. In both *npr-1(lf)* and the Hawaiian strain, we found an increase in locomotor quiescence among sleep-deprived animals relative to controls (Figure 6). This suggests that SIS is subject to homeostatic regulation and leads to the prediction that accumulated sleep pressure will limit the duration of arousal by oxygen. We see evidence of such a limit in some (Figures 1B and 3C) but not all (Figures 4C and 4D) of our SIS time courses, with a fraction of *npr-1(lf)* animals ultimately becoming quiescent. This increase in quiescence may be influenced by, but is not likely attributable to, changes in bordering and aggregation behavior throughout these assays (Figure S1).

## Discussion

An understanding of the core function of sleep and the mechanisms that allow for its coordination with competing needs is beginning to emerge through studies in model organisms. In *C*. *elegans*, exposure to damaging conditions triggers SIS that is beneficial for recovery, and animals tend to engage in more robust locomotor quiescence after, compared with during, exposure (Hill et al., 2014). Similarly, sleep in zebrafish larvae is associated with increased repair of DNA lesions, but quiescence sets in only following, not during, exposure to a DNA-damaging agent (Zada et al., 2019). These studies indicate that, although cellular SIS is conserved and critical for cellular repair, it can be delayed by the need to escape from aversive conditions. Our finding here that locomotor quiescence can be switched on and off during SIS indicates that quiescence can be overridden, not just delayed, by aversive stimuli.

Natural isolates of *C*. *elegans*, which live under reduced oxygen conditions associated with decomposing biomass such compost and rotting fruit, avoid higher oxygen concentrations associated with surface exposure (Persson et al., 2009). In this study, we find that hyperoxia promotes locomotor arousal from SIS, an effect that is dependent on primary oxygen sensors and TRPV channel activity, and is partly dependent on the arousal peptide PDF-1. This switch between behavioral states is rapid, sustainable for long periods, and fully reversible. The oxygen-sensitive sleep plasticity observable in a wild isolate is masked in the laboratory strain, N2, in part by a gain-of-function variant of the neuropeptide Y (NPY) receptor homolog NPR-1. Fortuitously, we can detect oxygen-dependent modulation of SIS in N2-derived *npr-1(lf)* animals, which we have isolated in a genetic screen for SIS-defective mutants.

**Figure 7:**
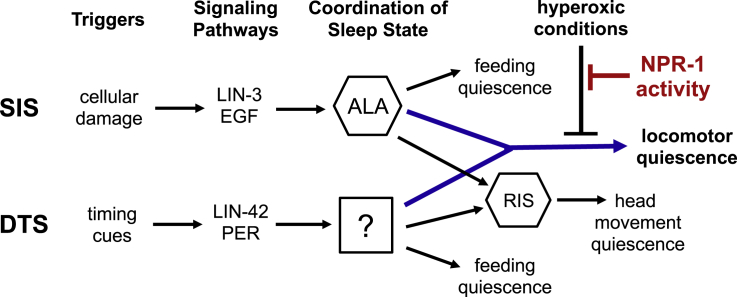
Model for Hyperoxia-Dependent Locomotor Arousal from Multiple Sleep States Behavioral quiescence during DTS and cellular stress-induced sleep (SIS) are initiated by different cues and mediated by distinct signaling pathways. During SIS, neuropeptides released by the ALA interneuron promote multiple sleep-associated behaviors, including reduced locomotion, head movement, feeding, defecation, and sensory responsiveness. It is not clear if a global sleep-promoting cell plays a similar role during DTS, but head movement quiescence in both states is dependent on the RIS interneuron. Locomotor quiescence in both DTS and SIS is rapidly reversed by hyperoxia when NPR-1 activity is low, suggesting that these sleep states also utilize a common effector of locomotor quiescence (blue arrow). Wild *C*. *elegans* isolates thrive in reduced-oxygen environments such as the inside of decomposing fruit, and hyperoxia is an indicator of surface exposure. Activation of oxygen-sensing neurons leads to secretion of arousal signals that override locomotor quiescence and allow escape. This oxygen-responsive plasticity in sleep is masked when NPR-1 activity is high (shown in red).

Similar to SIS, locomotor quiescence during DTS is rapidly reversed by hyperoxia avoidance in a manner that depends on NPR-1 (Choi et al., 2013, Nichols et al., 2017). This raises the possibility that locomotor quiescence in these two sleep states converges at a level that is highly plastic and subject to modulation by arousal cues (Figure 7). One potential target of modulation is the activity of the pre-motor “command neuron” network, which is highly synchronized in the awake state (Cho and Sternberg, 2014). The forward command neuron AVB is largely quiescent during DTS (Nichols et al., 2017) and is associated with higher calcium levels during forward locomotion at high oxygen than low oxygen (Laurent et al., 2015), indicating that modulation of AVB may contribute to oxygen-evoked arousal from quiescent states. Our finding that arousal depends partly on PDF-1 indicates that this peptide acts in parallel with yet-unidentified signals to arouse locomotion, a finding consistent with studies of PDF-1 function in DTS.

During SIS, the coordinated suppression of multiple active behaviors is mediated by the ALA interneuron, which promotes quiescence via the collective action of neuropeptides (NLP-8, FLP-13, FLP-24) with overlapping but distinct effects on the sub-behaviors of sleep, including feeding quiescence, head movement quiescence, and locomotion (Nelson et al., 2014; Nath et al., 2016). Interestingly, head movement quiescence during both SIS and DTS depends on the function of the RIS interneuron (Turek et al., 2013, Grubbs et al., 2019, Konietzka et al., 2019, Robinson et al., 2019). RIS-defective mutants, as well as animals with altered levels of ALA neuropeptides (Nath et al., 2016), reveal that active head movement does not override locomotor quiescence during SIS. There are no examples that we know of indicating that the converse is true, and it is possible that locomotor arousal is sufficient to promote side-to-side head movement. In the case of hyperoxia-aroused *npr-1(lf)* animals, which are active with respect to both head movement and locomotion (but not feeding), it may be that arousal cues act solely on the locomotor circuit.

Sleep homeostasis, which manifests as increased sleep amount or depth following sleep deprivation, indicates that sleep serves a function that cannot be bypassed (Cirelli and Tononi, 2008). However, certain arousal cues that have been found to suppress sleep also appear to reduce sleep need. For example, in *Drosophila* males, courtship-associated sleep loss is not associated with homeostatic sleep rebound, indicating that sexual arousal can counteract sleep pressure (Beckwith et al., 2017). Similarly, starvation-induced suppression of *C*. *elegans* SIS is not associated with the reduced viability normally associated with sleep loss under well-fed conditions (Goetting et al., 2018). The data presented here suggest that *C*. *elegans* SIS is subject to homeostatic regulation and that sleep pressure continues to accumulate in the hyperoxia-aroused state, an effect also apparent during DTS (Raizen et al., 2008, Nagy et al., 2014, Nichols et al., 2017). These data support the notion that sleep states in *C*. *elegans* serve critical functions. Although the mechanism remains unclear, SIS appears to enhance cellular repair, as sleepless ALA-impaired mutant animals show reduced survival after noxious heat exposure (Hill et al., 2014, Fry et al., 2016). This function may be common across species, as sleep in *Drosophila* promotes survival following heat exposure or infectious challenges (Kuo and Williams, 2014, Lenz et al., 2015), and sleep in zebrafish larvae promotes the repair of double-stranded breaks that accumulate during wakefulness (Zada et al., 2019). Interestingly, work in zebrafish has linked NPY signaling to an arousal-promoting system that impacts sleep-wake regulation (Singh et al., 2017), indicating that certain mechanisms that provide plasticity in sleep regulation may also be conserved between *C*. *elegans* and vertebrates.

### Limitations of the Study

Although the requirement for oxygen-sensing soluble guanylate cyclases and OSM-9 TRPV channel activity suggest involvement of the RMG sensory circuit, this study does not include cell-specific rescue experiments. Further analysis is required to identify the NPR-1 site of action in the modulation of SIS by hyperoxia.

## Methods

All methods can be found in the accompanying Transparent Methods supplemental file.

## Supplemental Information

Document S1. Transparent Methods and Figure S1

## References

[bib1] Anafi R.C., Kayser M.S., Raizen D.M. (2019). Exploring phylogeny to find the function of sleep. Nat. Rev. Neurosci..

[bib2] Beckwith E.J., Geissmann Q., French A.S., Gilestro G.F. (2017). Regulation of sleep homeostasis by sexual arousal. Elife.

[bib3] Bendesky A. (2012). Genetic variation in neurotransmitter receptors generates behavioral diversity. https://digitalcommons.rockefeller.edu/student_theses_and_dissertations/156.

[bib4] Chang A.J., Chronis N., Karow D.S., Marletta M.A., Bargmann C.I. (2006). A distributed chemosensory circuit for oxygen preference in *C. elegans*. PLoS Biol..

[bib5] Cheung B.H., Cohen M., Rogers C., Albayram O., de Bono M. (2005). Experience-dependent modulation of *C. elegans* behavior by ambient oxygen. Curr. Biol..

[bib6] Cho J.Y., Sternberg P.W. (2014). Multilevel modulation of a sensory motor circuit during *C. elegans* sleep and arousal. Cell.

[bib7] Choi S., Chatzigeorgiou M., Taylor K.P., Schafer W.R., Kaplan J.M. (2013). Analysis of NPR-1 reveals a circuit mechanism for behavioral quiescence in *C. elegans*. Neuron.

[bib8] Cirelli C., Tononi G. (2008). Is sleep essential?. PLoS Biol..

[bib9] de Bono M., Bargmann C.I. (1998). Natural variation in a neuropeptide Y receptor homolog modifies social behavior and food response in *C. elegans*. Cell.

[bib10] de Bono M., Tobin D.M., Davis M.W., Avery L., Bargmann C.I. (2002). Social feeding in *Caenorhabditis elegans* is induced by neurons that detect aversive stimuli. Nature.

[bib11] DeBardeleben H.K., Lopes L.E., Nessel M.P., Raizen D.M. (2017). Stress-induced sleep after exposure to ultraviolet light is promoted by p53 in *Caenorhabditis elegans*. Genetics.

[bib12] Fry A.L., Laboy J.T., Huang H., Hart A.C., Norman K.R. (2016). A Conserved GEF for Rho-Family GTPases acts in an EGF signaling pathway to promote sleep-like quiescence in *Caenorhabditis elegans*. Genetics.

[bib13] Goetting D.L., Soto R., Van Buskirk C. (2018). Food-dependent plasticity in *Caenorhabditis elegans* stress-induced sleep Is Mediated by TOR-FOXA and TGF-β signaling. Genetics.

[bib14] Gray J.M., Karow D.S., Lu H., Chang A.J., Chang J.S., Ellis R.E., Marietta M.A., Bargmann C.I. (2004). Oxygen sensation and social feeding mediated by a *C. elegans* guanylate cyclase homologue. Nature.

[bib15] Grubbs J.J., Lopes L.E., van der Linden A.M., Raizen D.M. (2019). A salt-induced kinase (SIK) is required for the metabolic regulation of sleep. bioRxiv.

[bib16] Hill A.J., Mansfield R., Lopez J.M.N.G., Raizen D.M., Van Buskirk C. (2014). Cellular stress induces a protective sleep-like state in *C. elegans*. Curr. Biol..

[bib17] Iannacone M.J., Beets I., Lopes L.E., Churgin M.A., Fang-Yen C., Nelson M.D., Schoofs L., Raizen D.M. (2017). The RFamide receptor DMSR-1 regulates stress-induced sleep in *C. elegans*. Elife.

[bib18] Isaac R.E., Li C., Leedale A.E., Shirras A.D. (2010). *Drosophila* male sex peptide inhibits siesta sleep and promotes locomotor activity in the post-mated female. Proc. R. Soc. B Biol. Sci..

[bib19] Jacobs B.L., McGinty D.J. (1971). Effects of food deprivation on sleep and wakefulness in the rat. Exp. Neurol..

[bib20] Keene A.C., Duboue E.R. (2018). The origins and evolution of sleep. J. Exp. Biol..

[bib21] Keene A.C., Duboué E.R., McDonald D.M., Dus M., Suh G.S.B., Waddell S., Blau J. (2010). Clock and cycle limit starvation-induced sleep loss in drosophila. Curr. Biol..

[bib22] Konietzka J., Fritz M., Spiri S., McWhirter R., Leha A., Palumbos S., Costa W.S., Oranth A., Gottschalk A., Miller D.M. (2019). Epidermal Growth Factor signaling acts directly and through a sedation neuron to depolarizes a sleep-active neuron following cellular stress. bioRxiv.

[bib23] Kuo T.-H., Williams J.A. (2014). Increased sleep promotes survival during a bacterial infection in Drosophila. Sleep.

[bib24] Laurent P., Soltesz Z., Nelson G.M., Chen C., Arellano-Carbajal F., Levy E., de Bono M. (2015). Decoding a neural circuit controlling global animal state in *C. elegans*. Elife.

[bib25] Lenz O., Xiong J., Nelson M.D., Raizen D.M., Williams J.A. (2015). FMRFamide signaling promotes stress-induced sleep in Drosophila. Brain Behav. Immun..

[bib26] MacFayden U.M., Oswald I., Lewis S.A. (1973). Starvation and human slow-wave sleep. J. Appl. Physiol..

[bib27] Macosko E.Z., Pokala N., Feinberg E.H., Chalasani S.H., Butcher R.A., Clardy J., Bargmann C.I. (2009). A hub-and-spoke circuit drives pheromone attraction and social behaviour in *C. elegans*. Nature.

[bib28] Monsalve G.C., Van Buskirk C., Frand A.R. (2011). LIN-42/PERIOD controls cyclical and developmental progression of *C. elegans* molts. Curr. Biol..

[bib29] Nagy S., Raizen D.M., Biron D. (2014). Measurements of behavioral quiescence in *Caenorhabditis elegans*. Methods.

[bib30] Nath R.D., Chow E.S., Wang H., Schwarz E.M., Sternberg P.W. (2016). *C. elegans* stress-induced sleep emerges from the collective action of multiple neuropeptides. Curr. Biol..

[bib31] Nelson M.D., Lee K.H., Churgin M.A., Hill A.J., Van Buskirk C., Fang-Yen C., Raizen D.M. (2014). FMRFamide-like FLP-13 neuropeptides promote quiescence following heat stress in *Caenorhabditis elegans*. Curr. Biol..

[bib32] Nichols A.L.A., Eichler T., Latham R., Zimmer M. (2017). A global brain state underlies *C. elegans* sleep behavior. Science.

[bib33] Persson A., Gross E., Laurent P., Busch K.E., Bretes H., de Bono M. (2009). Natural variation in a neural globin tunes oxygen sensing in wild *Caenorhabditis elegans*. Nature.

[bib34] Raizen D.M., Zimmerman J.E., Maycock M.H., Ta U.D., You Y.J., Sundaram M.V., Pack A.I. (2008). Lethargus is a *Caenorhabditis elegans* sleep-like state. Nature.

[bib35] Robinson B., Goetting D.L., Cisneros Desir J., Van Buskirk C. (2019). *aptf-1* mutants are primarily defective in head movement quiescence during *C. elegans* sleep. Micropubl. Biol..

[bib37] Singh C., Rihel J., Prober D.A. (2017). Neuropeptide Y regulates sleep by modulating noradrenergic signaling. Curr. Biol..

[bib38] Trojanowski N.F., Nelson M.D., Flavell S.W., Fang-Yen C., Raizen D.M. (2015). Distinct mechanisms underlie quiescence during two *Caenorhabditis elegans* sleep-like states. J. Neurosci..

[bib40] Turek M., Lewandrowski I., Bringmann H. (2013). An AP2 transcription factor is required for a sleep-active neuron to induce sleep-like quiescence in *C. elegans*. Curr. Biol..

[bib41] Van Buskirk C., Sternberg P.W. (2007). Epidermal growth factor signaling induces behavioral quiescence in *Caenorhabditis elegans*. Nat. Neurosci..

[bib42] Yurgel M.E., Kakad P., Zandawala M., Nässel D.R., Godenschwege T.A., Keene A.C. (2019). A single pair of leucokinin neurons are modulated by feeding state and regulate sleep–metabolism interactions. PLoS Biol..

[bib43] Zada D., Bronshtein I., Lerer-Goldshtein T., Garini Y., Appelbaum L. (2019). Sleep increases chromosome dynamics to enable reduction of accumulating DNA damage in single neurons. Nat. Commun..

