# Supplementary material for: NPR-1 Modulates Plasticity in C. elegans Stress-Induced Sleep
Source: iScience. 2019 Aug 30;19:1037–47. doi: 10.1016/j.isci.2019.08.050 (PMC6745490; doi:10.1016/j.isci.2019.08.050)
Supplement: Document S1. Transparent Methods and Figure S1 [file mmc1.pdf]

ISCI, Volume *19*

Supplemental Information

NPR-1 Modulates Plasticity in *C. elegans* Stress-Induced Sleep

Rony Soto, Desiree L. Goetting, and Cheryl Van Buskirk

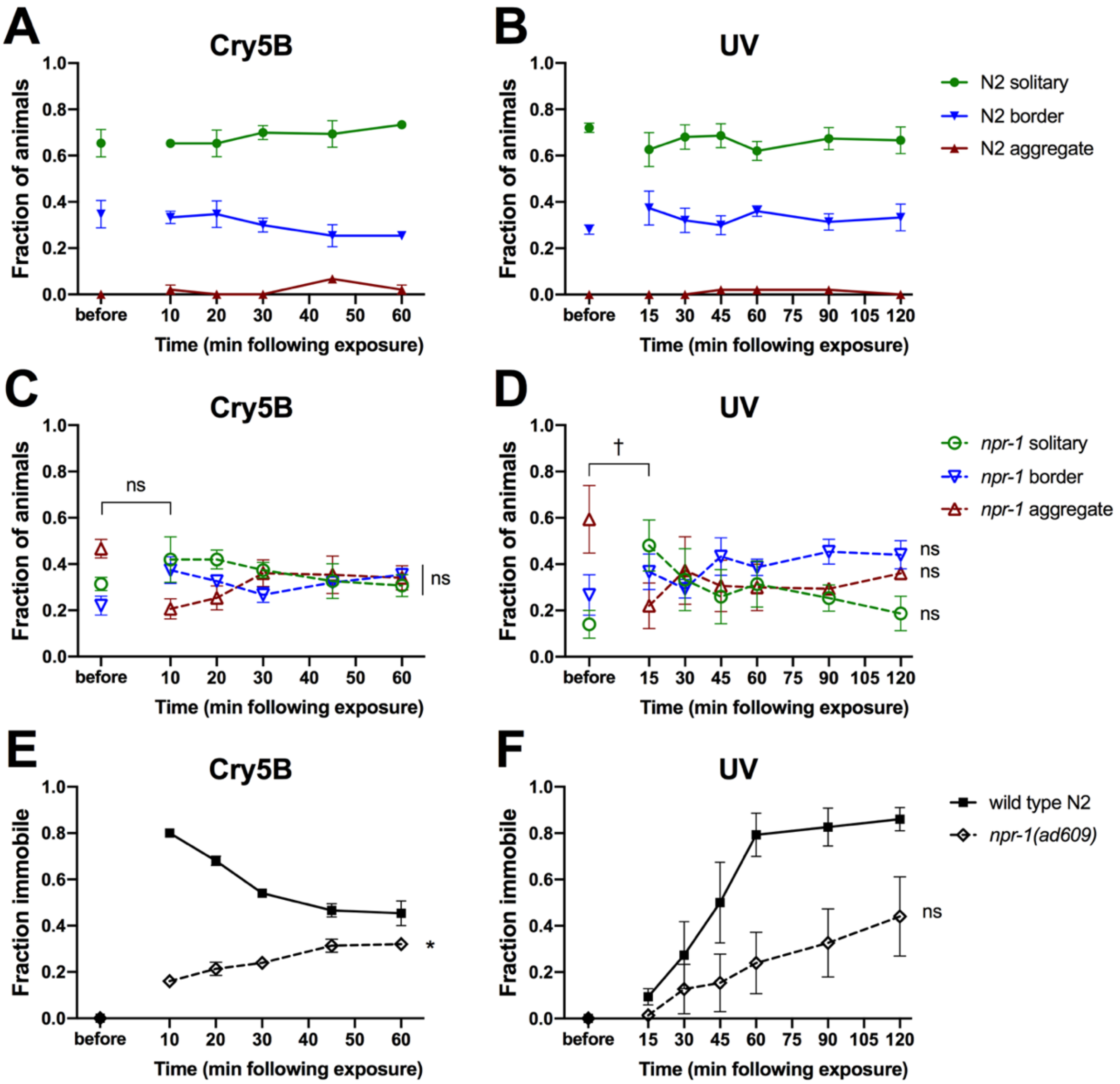

Figure S1. Quantification of bordering and aggregation behavior during time courses of stress-induced sleep (SIS), Related to Figure 4A. Wild-type N2 (A,B) and *npr-1(ad609)* mutant animals (C,D) were categorized as solitary, bordering, or aggregating, and also examined for immobility as a measure of stress-induced sleep (E,F). SIS was triggered by exposure to either pore-forming Cry5B toxin (A,C,E) or ultraviolet (UV) light (B,D,F) as described in Transparent Methods. Exposure to Cry5B toxin and UV light appear to impact the distribution of *npr-1(lf)* animals, an effect that is significant in the case of UV. ns = no significant differences, †P=0.031 fraction solitary before exposure vs. first time point, paired two-tailed t-test. Throughout the rest of each SIS time course, the distribution of *npr-1(lf)* animals does not change significantly (C,D). ns = not significant, one-way repeated measures ANOVA. However, the fraction of immobile *npr-1(lf)* animals appears to increase over time (E,F), an effect that is significant in the case of Cry5B. *P=0.032 one-way repeated measures ANOVA. These data suggest that exposure to SIS triggers can alter the distribution of *npr-1(lf)* animals, but that changes in distribution are not likely to account for the increased locomotor quiescence of *npr-1(lf)* mutants observed at later time points in some SIS assays.

TRANSPARENT METHODS

Nematode growth and staging Animals were raised under standard growth conditions at 20˚C on nematode growth media (NGM) plates seeded with *E. coli* OP50 unless otherwise noted. All experiments were performed on well-fed young adults (post - L4 molt animals in which eggs were not yet visible in the uterus) that had been examined for regular movement and pharyngeal pumping prior to manipulation.

Behavior measurements and statistical analysis Behavior was examined under a stereomicroscope, with plates gently slid into the field of view 45 sec prior to examination, with the exception of the undisturbed assay (Figure 3D), in which case each plate remained in the field of view of its own stereomicroscope prior to examination. Each animal was observed for 5 sec in the determination of quiescence, and at least 25 animals were examined per trial to determine the fraction quiescent. At least three independent trials were performed for each experiment. ALA-defective *ceh-17(lf)* mutant animals were used to identify time windows of ALA-dependent quiescence following exposure to each SIS trigger. Locomotor quiescence was defined as complete immobility including the absence of head movement, and feeding quiescence as a lack of pharyngeal pumping. An animal displaying any of these active behaviors was categorized as non-quiescent. Where feasible, the experimenter was blind to genotype and condition. Statistical tests, indicated in all figure legends, were performed using Prism 7 software.

Examination of behavior in the absence of food (Figures 1C and 1D) Animals were rinsed 3 times in M9, pipetted to peptone-free NGM plates that were either seeded with *E. coli* OP50 (+ food control) or not, exposed to SIS triggers (or mock-handled), and examined for SIS as well as locomotor activity, quantified as the number of body bends observed in one minute of observation. A body bend was defined as the completion of a peak-to-peak movement of sinusoidal locomotion in the forward or reverse direction.

UV exposure Animals were placed on seeded NGM plates, which were placed lid-side down on a 302 nm (UV-B), 60 mW/cm^2 ultraviolet light source for 50 sec. Under these conditions, robust SIS lasts for several hours beginning approximately 45 min after exposure (see Figures 3C and 4D). For assays involving single-time point analysis of UV-SIS, the 60 min time point was chosen.

Cry5B exposure Animals were placed onto NGM plates containing Cry5B-expressing bacteria as previously described (Hill et al., 2014) and examined for SIS after 15 min of exposure. For time courses (Figures 1B and 4C), this initial scoring on Cry5B represents the zero time point, after which animals were transferred to OP50-seeded plates for subsequent time points. For examination of Cry5B-SIS under low-oxygen conditions, a 10 min exposure was used instead of the standard 15 min to allow time for subsequent manipulation prior to examination.

Osmotic stress (Figure 2A) Animals were placed on NGM plates containing 500mM NaCl (regular NGM contains 50 mM NaCl) and *E. coli* OP50. Animals were exposed for 20 min, transferred to seeded regular NGM plates and examined 30-40 min later for locomotor and feeding quiescence**.**

Heat stress (Figure 2B) Animals were placed on seeded 60 mm NGM plates, which were sealed with parafilm, placed upright in a 37°C water bath for 11 min and transferred to ice for 2 min to cool to room

temperature. Under these conditions, ALA-dependent sleep peaks at 10-20 min after heat shock (Goetting et al., 2018).

Transgenic EGF overexpression (Figure 2C) To induce EGF expression from the syIs197 integrated hs:LIN-3 transgene in both wild-type and *npr-1* mutants, 60 mm seeded NGM plates containing transgenic animals were parafilmed, placed into a 33˚C water bath for 30 min to trigger expression from the heat shock promoter, and examined 2 hr later for EGF-induced quiescence. These induction conditions also produce a transient heat-SIS response (Nelson et al., 2014) that terminates before the time that EGF(OE)-induced sleep is examined.

Controlled oxygen environment (Figures 4B and 6) A 7.6 cm x 3.8 cm (3 in x 1.5 in) clear Hammond chamber with 6.4 mm (¼ in) holes drilled at the long ends was used to house two 35 mm diameter (4 ml) NGM plates containing young adult animals to be examined for SIS under controlled oxygen conditions. Size 0 rubber stoppers were used to plug each end, and one of these was drilled with a 1.6 mm (1/16 in) hole for gas delivery. The wide end of a P200 pipet tip was inserted into 3.2 mm (1/8 in) nylon tubing and sealed with parafilm at the junction, and the small end was inserted through the rubber stopper. The tubing was fitted to a Harris regulator on a 10% oxygen, 90% nitrogen tank (Airgas). Animals to be examined for SIS were exposed to UV light or Cry5B toxin as described above and placed into the chamber. The chamber lid was sealed with vacuum grease (Super Lube) and parafilm, and placed on a stereomicroscope for examination of behavior. To lower the oxygen concentration, the chamber was flushed three times with the low-oxygen mix and then stoppered, and a process that took roughly 15 seconds. To return to ambient (21%), the stoppers were simply removed, allowing ambient air to fill the chamber. Each transition was made without movement of the chamber.

Bordering and aggregation (Figure 4A) Lawns were produced by placing 5 x 30µl spots of a 50% glycerol solution spaced out on a 60 mm NG plate and allowed to dry, followed by addition of 30µl of OP50 to each spot and allowed to grow for 4 days to create thick lawns of OP50 approximately 10 mm in diameter. Enough OP50 solution to just cover the surface was added and allowed to dry overnight, producing a thin lawn in between the thick lawn areas. Animals were transferred to these plates, exposed to UV light or mock handled, left at 20˚C for one hour and examined for UV-SIS as described above. Aggregating behavior was defined as direct contact along at least 50% body length among at least three animals. While sleep behavior can be difficult to assess in large aggregates, the aggregates here contained between 3-8 adults and SIS was observable under a stereomicroscope. Bordering behavior was measured by those animals with at least their head inside the thick bacterial lawn. Animals that were neither aggregating nor bordering were defined as solitary.

Bordering, aggregation, and locomotor quiescence during SIS time courses (Figure S1) To assess bordering and aggregation behavior in the context of our standard stress-induced sleep (SIS) time courses, animals were examined on regular NGM plates seeded 3 days prior with *E. coli* OP50 resulting in a bacterial lawn approximately 30 mm in diameter with a thick border. Following transfer, animals were left undisturbed for 15 min and examined for bordering and aggregation behavior prior to exposure to conditions that trigger SIS. For Cry5B SIS, animals were transferred to plates seeded with Cry5B-expressing bacteria and exposed for 15 min before return to NGM plates described above. For UV-SIS, plates were simply placed lid-down on a 60mW UV light box and exposed for 50 sec. Bordering and aggregation were defined as above, and immobility was defined as showing no detectable movement during a 5 sec observation under the stereomicroscope.

Data availability The raw datasets corresponding to Figures 1-6 and Figure S1 are publicly available at Mendeley Data https://data.mendeley.com/datasets/x4p68hxt46

Strains used in this study

**Strain**	**Description**	**Source**
N2	Wild type, Bristol isolate (standard laboratory strain).	CGC
CB4856	Wild type, Hawaiian isolate.	CGC
CVB28	syIs197 V; *npr-1(ky13)* X	This study
CVB32	*npr-1(csn7)* X.	This study
CX10	*osm-9(ky10)*	CGC
CX4148	*npr-1(ky13)* X.	C. Bargmann
CX4821	*osm-9 (ky10)* IV; *npr-1(ad609)* X	C. Bargmann
CX6448	*gcy-35(ok769)*	CGC
CX7157	*gcy-35 (ok769)* I; *npr-1(ad609)* X	C. Bargmann
CX11400	kyIR9 [X: ~4745910 - ~4927296, N2>CB4856] X	CGC
DA609	*npr-1(ad609)* X	CGC
IB16	*ceh-17(np1)* I	CGC
KP6100	*pdf-1(tm1996)* III*; npr-1(ky13)* X	J. Kaplan
LSC27	*pdf-1(tm1996)* III	CGC
PS5970	him-5(e1490) syIs197 V	CGC
**Transgene**		
syIs197	hs::LIN-3c(cDNA) + myo-2p::DsRed + pha-1(+) V	CGC
